# The recognition of oral manifestations of haematological disease saves lives: a case report

**DOI:** 10.1186/s42269-022-00915-9

**Published:** 2022-09-03

**Authors:** Fabienne Aurora, Anita Arasaretnam, Andrew Hobkirk

**Affiliations:** 1 Bristol Dental Hospital, Lower maudlin street, Bristol, BS1 2LY UK; 2 Royal Sussex County Hospital NHS Trust, Brighton, UK

**Keywords:** Oral surgery, Diagnosis, Leukaemia, Oral manifestations, COVID-19

## Abstract

**Background:**

Acute Leukaemias are haematological disorders characterised by the proliferation of immature white blood cells in the bone marrow and/or peripheral blood. Oral manifestations of leukaemia are common and may be the first sign of the disease. The clinical presentation of these Acute Leukaemias may include neutropenic sepsis, hyperviscocity and coagulopathy which confer a potential morbidity and mortality. Clinicians must be able to recognise this pattern of presentation.

**Case report:**

We report a 34-year-old female who was referred to the Oral and Maxillofacial Surgery department with acute dental pain and pericoronitis. She subsequently had a simple dental extraction but re-presented with a bleeding socket that did not respond to local treatment. Investigation of this led to a diagnosis of Acute Promyelocytic Leukemia (APL). She was admitted under the care of the haematology team for urgent, life-saving, treatment.

**Conclusions:**

Early diagnosis and treatment of the Acute Leukaemias can be life saving. The oral manifestations of disease are common and may be the first sign. Clinicians must be able to recognise this pattern of presentation and arrange urgent investigation and specialist management.

**Clinical/CPD relevance:**

This case report discusses leukaemia and highlights the important role General Dental Practitioners can play in early diagnosis. We frame a safe approach to managing these patients in a typical case. Whilst this disease subtype is rare, the learning points can be universally applied.

## Background

Leukaemia is a malignant proliferation of immature leukocytes in the bone marrow and or blood, with the first case description being published in 1845 by John Hughes Bennet. He described hypertrophy of the spleen and liver in which death took place from suppuration of the blood (Bennett 1845). The oral manifestations associated with Acute Leukaemias have been widely reported (Rosa et al. 2018). The updated WHO classification of haematological malignancies classifies Leukaemia into chronic and acute subtypes: lymphoid or myeloid (Arber et al. 2016). Acute Myeloid Leukaemia (AML) is an aggressive disease which is rarely diagnosed before the age of 40 years and has a slight male predominance in most countries (Guan and Firth 2015). Acute Promyelocytic Leukaemia (APL) is a malignant subtype of AML and has a prevalence of approximately 10–15% in adults (Soignet et al. 1998) with AML.

General Dental Practitioners (GDP) may be the first health care professionals to identify the oral manifestations of leukaemia, and it is essential that they recognise their significance. Oral signs include gingival hyperplasia, spontaneous gingival bleeding, petichae and candida infections. These can all easily be erroneously attributed. We highlight the importance of pattern recognition, baseline blood tests and the timely referral of these patients to specialist services.

## Case presentation

A previously healthy 34-year-old female of Asian ethnicity completed a telephone consultation with her GDP following national guidelines during the COVID-19 pandemic. She gave a 5-day history of ‘spontaneous gum bleeding, gum swelling, dental pain from her left upper wisdom tooth and swelling below the lower jaw’. She had recently developed a fever and had limitation of mouth opening to ‘one finger breadth’.

The diagnosis from this telephone consultation was ‘severe periocoronitis or periodontal abscess associated with the upper left wisdom tooth’. She was managed following the Scottish Dental Clinical Effectiveness Programme Advice, Analgesic and Antimicrobial guidance (introduced during the COVID-19 pandemic to optimise triage) with paracetamol and ibuprofen and a prescription for amoxicillin and metronidazole. Thirteen days later she telephoned the same clinician to discuss her on-going symptoms. These included gum swelling, cheek swelling, gum bleeding, a lump in her right throat, unexplained weight loss and constipation. This triggered a two week wait (2WW) referral to the Oral and Maxillofacial Surgery (OMFS) department.

### Investigations

The patient was seen on the OMFS 2WW clinic at the Royal Sussex County Hospital (RSCH) 9 days later.

She presented with pain and gingival swelling around the lower right wisdom tooth which had not responded to her oral antimicrobials. Her mouth opening had improved to 25 mm measured intra-incisally.

**Fig. 1:**
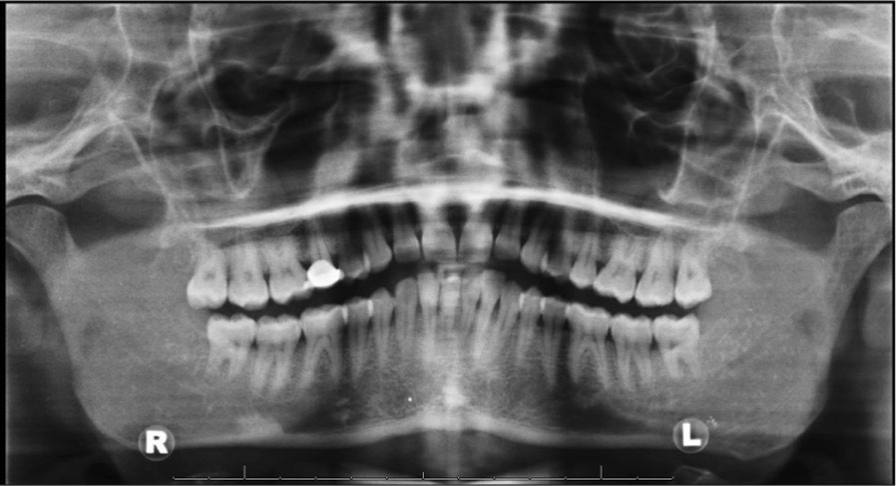
Orthopantomogram taken at RSCH 2WW clinic showing partially over-erupted UR8

On clinical examination, the patient had bilateral posterior mandible pain and right mandibular gingival hyperplasia. An Orthopantomogram (OPG) shown in Fig. 1 confirmed the tooth anatomy and did not show any significant dental pathology.

### Differential diagnosis

A diagnosis of lower right third molar gingival hyperplasia and pericoronitis was made.

She declined excision of the LR8 operculum under local anaesthesia at this appointment and was given a 7-day course of oral co-amoxiclav with a review appointment booked the following week.

At review in the OMFS department the oral signs had not changed. The patient reported new blurred vision and was urgently referred to the Ophthalmology Department, Sussex Eye Hospital, where a diagnosis of sub-inner limiting membrane haemorrhage of the left eye was made. This was managed ‘conservatively’.

The patient had a simple extraction of the over-erupted UR8 and an operculectomy LR8 under local anaesthesia the following week. There were no immediate complications. Further systemic symptoms were identified: lethargy, unanticipated weight loss and she had not opened her bowels for nearly two weeks. She had not been prescribed opiate analgesia and her GP was investigating her symptoms. Her weight loss had been attributed to reduced calorie intake due to oral pain.

### Hospital admission

The patient attended the RSCH Emergency Department the following day with continuous bleeding from the extraction site and severe abdominal pain. She was stabilised and had a number of blood tests. Relevant abnormal results are shown in Table 1.

**Table 1:** FBC, Blood Film, Coag, Fib, D-dimer, CRP, Ferritin, LDH

Full blood count and blood film			
Haemoglobin	41	g/L	115–165
White cell count	9.7	10^9^/L	4–10
Neutrophil count	0.3 3.0%	10^9^/L	2–7
Platelet count	18	10^9^/L	150–410
Haem consultant blood film comment: morphology consistent with APL (hypergranular promyelocytes)—urgent haem review arranged			
*Clotting screen*			
INR	1.6		0.8–1.2
APTT	40.8	secs	24.8–37.4
APTT ratio	1.38		0.87–1.15
Prothrombin time	22.9	secs	11–17
Fibrinogen	1.6	g/L	1.8–4.5
*Other*			
D-Dimers assay	> 20.00	ug/mL	0–0.5
Serum C-reactive protein	105	mg/L	0–5
Serum ferritin	694	ug/L	13–150
Serum LDH	998	iu/L	240–480

**Fig. 2:**
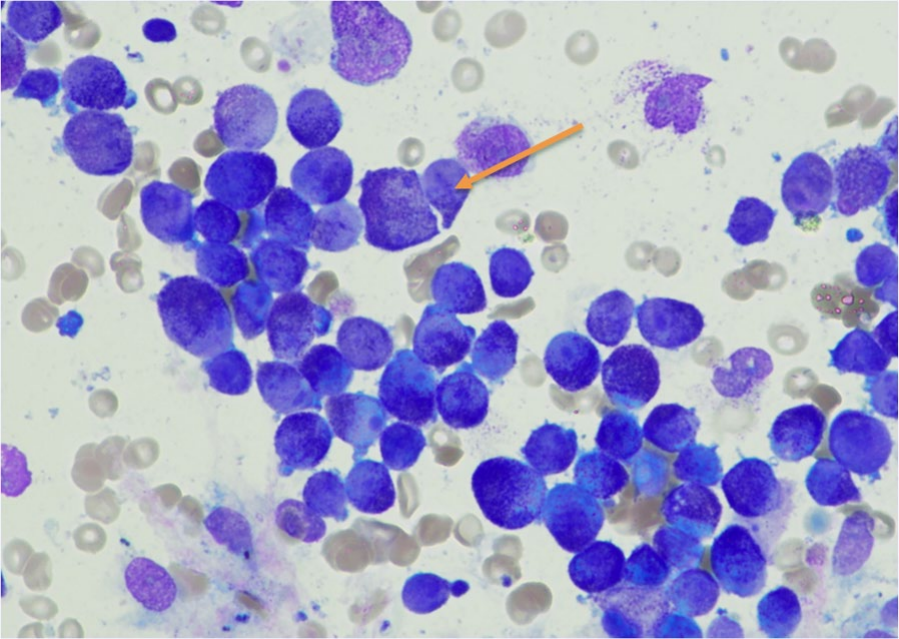
Diagnostic Bone Marrow Aspirate (× 50 Power): Hypercellular marrow, abnormal promyelocytes with azurophilic granulation, some containing Auer rods (arrow)

On the basis of this clinical/blood picture and blood film morphology (Fig. 2), a diagnosis of APL was made, and she was reviewed immediately by the Haematology Department and admitted under their care.

Presentation bloods show a marked pancytopenia with microscopic examination of a blood film highlighting the presence of blasts and hypergranular promyelocytes, with morphological features of those seen in APL. The clotting screen highlights a Disseminated Intravascular Coagulation (DIC) picture with an elevated PT and APTT, a low Fibringen and elevated D-Dimers. Acute phase reactants including serum ferritin and C-reactive protein were elevated, along with the serum LDH – a ubiquitous, non-specific marker of cell turnover.

Bone marrow examination identified the classical Promyelocytic Leukemia Retinoic Acid Receptor Alpha *(PML- RARA)* fusion sequence chromosomal translocation t(15;17), confirming the diagnosis of APL. Further investigations were undertaken to determine the nature of the abdominal pain and change in bowel habit with an MRI of the ano-rectal region and surgical opinion, confirming a diagnosis of a fistula which was managed conservatively because of the patient’s neutropenia.

### Treatment

Immediate treatment focussed on correcting the coagulopathy with platelet transfusion to maintain platelet count > 30–50 × 10^9^/L and fresh frozen plasma and cryoprecipitate to normalise the PT and APTT and to achieve fibrinogen levels > 1.5 g/L (Sanz et al. 2009; Tallman and Altman 2009).

Red Blood Cell transfusions were administered to correct the profound anaemia. Broad spectrum antimicrobials were commenced to cover translocation of gastrointestinal tract organisms along with other prophylactic antiviral and antifungal medications, because of risk in a neutropenic presentation.

AllTrans-Retinoic Acid (ATRA) was commenced upon immediate clinical suspicion of APL, before subsequent confirmation of the diagnosis. This acts to differentiate the abnormal promyelocytes and ultimately the cells undergo apoptosis. It also helps to reduce tissue factor secretion by the abnormal promyelocytes which otherwise drives the DIC picture (Fenaux et al. 1993).

Within 24 h of starting ATRA, the patient became pyrexial, breathless and hypoxic. Transfer to the Intensive Care Unit was necessary and a clinical concern regarding a recognised cardiorespiratory complication of APL, namely differentiation syndrome, was managed with cessation of the ATRA and administration of IV Dexamethasone (Sanz et al. 2009).

Cytoreduction with oral hydroxycarbamide chemotherapy was commenced to control the proliferative WCC. The ATRA was slowly reintroduced at a later date, once the patient had improved from a cardiorespiratory view point.

First line treatment was commenced with Arsenic Trioxide (ATO) induction. The ATO, in combination with ATRA is a non-chemotherapy option for patients with so called ‘low-risk’ disease—in this case due to the presentation WCC of < 10 × 10^9^/L (Overview 2021).

The patient returned back to the haematology ward for the rest of her inpatient care. Regular monitoring was required for ATO complications including QTc prolongation, with judicious electrolyte correction and caution regarding the addition of any QTc prolonging medications including anti-emetics.

Ophthalmological inpatient reviews were obtained to assess the patients visual blurring, noted pre-admission. Examination confirmed a unilateral retinal haemorrhage, managed expectantly but with a higher initial target platelet threshold of 100 × 10^9^/L for two weeks.

The oral biopsy, taken pre-admission from the operculum, was found to have mucosal infiltration by immature myeloid cells.

Bone marrow examination after this first induction cycle of treatment documented a morphological remission with a very low level of the *PML-RARA* being detected by sensitive molecular techniques.

As an outpatient, blocks of ATRA and ATO consolidation have been delivered over a nine-month period.

Subsequent bone marrow examinations have documented ongoing morphological remission and no evidence of any molecular minimal residual disease.

### Outocome and follow up

The oral infiltration and ano-rectal fistula healed conservatively, aided by the restoration of the patient’s own neutrophils.

The patient’s pre-retinal haemorrhage improved spontaneously with almost complete reabsorption of the haemorrhage and normal visual acuity being restored.

At the most recent follow up, ten months post initial diagnosis, the patient was alive and well. Her end of treatment bone marrow assessment confirmed her to be in remission. She has regular follow up in the haematology clinic.

## Discussion

AML is a heterogeneous disorder categorised by bone marrow failure and the proliferation of immature myeloid cells. It is the most common acute leukaemia among adults (Saultz et al. 2016; De Kouchkovsky and Abdul-Hay 2016). APL is a subtype of AML with both unique morphological and clinical presentations (Cicconi and Lo-Coco 2016; McCulloch et al. 2017). The 2016 WHO classification of myeloid neoplasms and myeloid malignancies identifies *PML-RARA* to be associated with the sub-type APL, making this the specific characteristic (Arber et al. 2016).

AML and APL present differently, have different cure rates and different treatment approaches. Treatment outcomes for non-promyelocytic AML and APL are different. Standard AML has a cure rate of 60% in younger patients, compared to 10–15% (Burnett 2005) in those over 60 years. APL does not tend to have the coagulopathy of an APL presentation, with the associated early bleeding mortality. With current therapy, APL is the subtype of AML with the highest potential cure rate of 80–90% (Tallman et al. 2002).

APL itself is regarded as a haematological emergency presentation. It has a high morbidity and mortality in the early stages because of the associated DIC. Clinical sequelae including life threatening bleeding and thrombosis may occur. Up to 40% of patients may develop a central nervous system or pulmonary haemorrhage with a 10–20% incidence of haemorrhagic death (Rodeghiero et al. 1990; Lehmann et al. 2011). Many patients die of bleeding complications before even presenting to specialist teams and so the window of opportunity for early recognition and successful treatment is short, with early referral to a specialist service vital.

The treatment involves ATRA plus ATO or ATRA plus anthracycline containing chemotherapy, depending on the patients WCC stratified risk group (Platzbecker et al. 2017; Sanz et al. 2010).

The differentiation syndrome seen in APL may consist of clinical features including unexplained fever, weight gain, and shortness of breath, respiratory infiltrates/effusions, hypotension and renal failure. Delayed recognition of this condition with its many clinical differentials is a potential pitfall. Guidelines suggest management with corticosteroids and cessation of the ATRA or ATO in severe cases (Sanz and Montesinos 2014).

In leukaemia, malignant immature white blood cells proliferate at the expense of erythrocytes, resulting in anaemia, fatigue, pallor and weakness. The platelet count drops resulting in gingival bleeding and petechiae (Arora et al. 2020). Immunosuppression leads to oral ulceration, bacterial, viral and fungal infections and oral candidiasis is common (Stafford et al. 1980). Gingival swelling is thought to be associated with leukemic infiltration, especially in patients with a high white blood cell count (Hou et al. 1997).

### Ophthalmic manifestations

Leukaemia may involve ocular tissues via a number of mechanisms including direct infiltration, haemorrhage, ischaemia and chemotherapeutic agent toxicity reactions. There is an increased rate of viral, fungal, protozoal and bacterial eye infections during immunosuppression and patients are at risk of graft versus host disease during allogenic bone marrow transplantation.

In this patient a diagnosis of sub inner limiting membrane haemorrhage of the left eye was confirmed when the patient presented with sudden onset altered vision in her left eye. Retinal haemorrhages are one of the most conspicuous eye signs of leukaemia and causes include thrombocytopenia, anaemia, hyper-viscosity and immunosuppression. Eye signs in leukaemia may signify extramedullary disease and timely ophthalmological assessment of these is essential (Sharma et al. 2004).

### Post-extraction bleeding

Tooth extraction causes trauma with bleeding from the alveolar bone and soft tissues. This is normally readily controlled within minutes with local measures, such as direct pressure, until a blood clot has formed. Prolonged bleeding may be primary, reactionary or secondary. Lockhart (2003) defined post-extraction bleeding (PEB) as bleeding which:

1. Continues beyond 12 hours;
2. Causes the patient to call or return to the dental practitioner, or attended an accident and emergency department;
3. Results in the development of a large haematoma or ecchymosis within the oral soft tissues; or
4. Requires a blood transfusion, hospitalisation, or both.

The incidence of PEB following extraction of maxillary third molars is 0.4% and for mandibular third molars it is 0.6% (Chiapasco et al. 1993). PEB is related to local and systemic factors. Local factors include tooth site, concurrent local infection, blood vessel injury, more traumatic extractions and poor patient compliance with post-operative wound care instructions. Systemic factors include haematological deficiencies or abnormalities, diseases which affect coagulation and the use of anti-coagulant and antiplatelet medication.

## Conclusions

The oral manifestations of leukaemia are a well-known, documented, phenomenon and often present early in disease progression. Patients with leukaemia can deteriorate rapidly and dentists have an important role to play in disease recognition and urgent referral for specialist management. The SARS COV 2 pandemic put unique pressures on the delivery of dentistry. With a limited number of face to face appointments available telephone triage became an important tool. This case highlights the limitations of telephone triage in a patient who, ultimately, was shown to be presenting with a life threatening haematological disease.

## Key take home messages

Key messages from this paper:

- Recognition of the oral and head and neck signs of leukaemia can lead to early diagnosis
- Early diagnosis and treatment of APLis related to better patient outcomes because APL rapidly causes potentially fatal coagulopathy
- General dental practitioners’ must appropriately screen patients for the oral and head and neck signs of systemic disease

*Think* Does my patient need a blood test?

## Data Availability

Not appropriate.

## Abbreviations

OMFS: Oral maxillofacial surgery
APL: Acute Promyelocytic Leukaemia
AML: Acute myeloid leukaemia
RSCH: Royal Sussex County Hospital
PML-RARA: Promyelocytic leukemia retinoic acid receptor alpha
WCC: White cell count
QTc: Corrected QT interval
GDP: General Dental practitioner
ATRA: All-trans-retinoic acid
ATO: Arsenic Trioxide
DIC: Disseminated intravascular coagulation
PEB: Post-extraction bleeding
